# An Excel‐based implementation of the spectral method of action potential alternans analysis

**DOI:** 10.14814/phy2.12194

**Published:** 2014-12-11

**Authors:** Charles M. Pearman

**Affiliations:** 1 Institute of Cardiovascular Research, The University of Manchester, 3rd Floor, Core Technology Facility, Grafton Street, Manchester, M13 9XX, U.K

**Keywords:** Action potential, alternans, Excel, Fourier, spectral, Visual Basic for Applications

## Abstract

Action potential (AP) alternans has been well established as a mechanism of arrhythmogenesis and sudden cardiac death. Proper interpretation of AP alternans requires a robust method of alternans quantification. Traditional methods of alternans analysis neglect higher order periodicities that may have greater pro‐arrhythmic potential than classical 2:1 alternans. The spectral method of alternans analysis, already widely used in the related study of microvolt T‐wave alternans, has also been used to study AP alternans. Software to meet the specific needs of AP alternans analysis is not currently available in the public domain. An AP analysis tool is implemented here, written in Visual Basic for Applications and using Microsoft Excel as a shell. This performs a sophisticated analysis of alternans behavior allowing reliable distinction of alternans from random fluctuations, quantification of alternans magnitude, and identification of which phases of the AP are most affected. In addition, the spectral method has been adapted to allow detection and quantification of higher order regular oscillations. Analysis of action potential morphology is also performed. A simple user interface enables easy import, analysis, and export of collated results.

## Abstract (precis)

Action potential alternans is mechanistically linked to cardiac arrhythmogenesis and sudden cardiac death. A software tool is presented here to facilitate alternans quantification.

## Introduction

**Figure 1.**
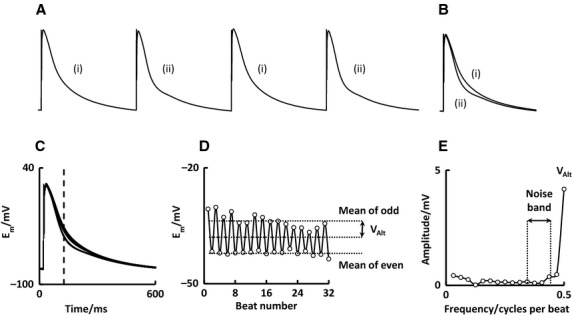
Analysis steps for a typical cell. (A) Nonaligned action potentials. (B) Beat‐aligned average action potentials highlighting alternans. (C) 32 superimposed action potentials. Dashed line represents timepoint showing maximal V_A__lt_. (D) E_m_ at each beat from timepoint in A. (E) Magnitude spectrum of B.

The cardiac action potential (AP) is the pattern of depolarization and repolarization of the cell membrane of cardiac myocytes with every heartbeat. Under normal conditions, repetitive stimulation of a myocyte generates action potentials that are similar from one beat to the next. AP alternans is the observation that under certain conditions the shape of the AP varies on a beat by beat basis between two contrasting states (Fig. 1A and B). This may take the form of AP duration alternans, that is, long‐short‐long‐short or AP amplitude alternans, that is, large‐small‐large‐small (Myles et al. 2011).

It has been recognized for more than a century that alternation of cardiac output heralds a grave prognosis (Traube 1872) and that this is connected with alternation of cardiac repolarization (Lewis 1910). More recently, electrical alternans at the whole‐heart scale has been linked to alternans at the cellular level (Narayan et al. 2008a). Action potential alternans is associated with abnormal heart rhythms affecting the cardiac atria (Narayan et al. 2008b), the ventricles (Myles et al. 2011), and the likelihood of sudden cardiac death (Laurita and Rosenbaum 2008).

The original description of alternans was of a variation in cardiac output, corresponding to ventricular depolarization. Conversely, T‐wave alternans corresponding to ventricular repolarization is the most widely studied aspect of alternation of cardiac electrical activity. Both depolarization and repolarization alternans are associated with vulnerability to arrhythmias (Gordon et al. 2010), and these facets of alternans behavior can both occur at different times (Wang et al. 2014), and exist in or out of phase with each other (Jing et al. 2012). It therefore follows that it may be useful to examine alternans of each region of the action potential independently.

Although the alternating pattern of 2:1 oscillations has been most widely studied, descriptions of higher order periodicities including 3:1, 4:1 and more complex rhythms have also been described (Bien et al. 2006). It has been suggested that these more complex patterns may represent a further step toward chaotic fibrillation (Nearing and Verrier 2002). Identification of these patterns may therefore be of great importance in the assessment of a pro‐arrhythmic substrate.

In order to understand the mechanisms of alternans or the conditions that precipitate it, a robust method of alternans quantification is required. While gross alternans of clean signals can be easily seen using the naked eye, subtle alternans or analysis of signals contaminated by noise requires more sophisticated detection methods. The spectral method of alternans analysis, first described by Adam et al. (1984), is one method used for the study of AP alternans (Lalani et al. 2013; Wang et al. 2014). This technique is also very well established for the analysis of the related phenomenon of T‐wave alternans on the surface ECG (Verrier et al. 2011).

Commercially available and open‐source tools (Khaustov et al. 2008) can be used to assess T‐wave alternans, but these are unsuitable for the interpretation of AP alternans. No software exists in the public domain for AP alternans analysis as previously published work in this field has used custom written software that has not been made accessible to the wider academic community.

This study represents the first software to be made freely available to aid the analysis of AP alternans. It describes a program written in Visual Basic for Applications (VBA) to run within Microsoft Excel. Excel is used as a shell and the user is not required to engage with the fundamental processes involved. This approach, using a commonly available spreadsheet package adapted with the VBA language to become a tool for signal processing, has been adopted previously for analyzing other aspects of cardiac electrophysiology (Greensmith 2014). An advantage of this method is that every run can be saved as a separate Excel workbook for future reference complete with easily exported graphs. Although the program is tailored for AP alternans, it can be applied to the study of alternans of other parameters such as calcium transient alternans by bypassing the “AP morphology” processing step as discussed below.

## Methods

A detailed analysis of alternans behavior should accomplish several aims; (1) the presence or absence of alternans should be determined, crucially discriminating alternans from random fluctuations between beats, (2) the magnitude of alternans if present should be quantified, (3) the regions of the APs affected should be analyzed independently, (4) higher order oscillations such as 3:1 or 4:1 should be identified.

Spectral analysis accomplishes these aims by converting a train of APs recorded in the time domain to a series of frequencies using a discrete Fourier transform (DFT). The frequencies in this case represent how rapidly a parameter changes between beats. They are the reciprocal of the number of beats a regular oscillation takes to return to its starting value. For example, a parameter that cyclically changes in a pattern A,B,C,A,B,C,A… has a period of three beats/cycle and therefore has a frequency of 0.33 cycles/beat. The DFT assesses all possible frequencies in the series (limited by sample size) and identifies the extent to which each frequency is present. In the frequency domain, 2:1 oscillations specific to alternans occur at a frequency of 0.5 cycles/beat. This can be separated from nonregular oscillations (noise) or higher order regular oscillations. The magnitude of alternans (*V*_Alt_) can be determined from the height of the peak at 0.5 cycles/beat.

### Discrete Fourier transform

A train of action potentials such as is shown in Fig. 1A is beat aligned so that the upstroke of each beat falls at the same timepoint (Fig. 1B). Commercially available electrophysiology recording software such as Clampex (Molecular Devices) automatically records sequential traces that are aligned to stimulation time and this function is not duplicated here. Care must be taken when aligning signals in the y (voltage) axis as inappropriate trace alignment can lead to a distortion of alternans quantification.

A matrix is constructed whereby the first column of data is a timebase, while the second and subsequent columns represent membrane potential (*E*_m_) at that timepoint for each beat in turn. Plotting the matrix as time on the *x*‐axis and *E*_m_ on the *y*‐axis yields superimposed action potentials such as in Fig. 1C.

*E*_m_ can be plotted at the same timepoint for each beat as shown by the dotted line in Fig. 1C, resulting in Fig. 1D. This visualizes how *E*_m_ at the same timepoint changes across beats.

A DFT is used to convert data in the time domain to data in the frequency domain. It makes use of equation 1 (Peters et al. 1998). The underlying principle is that any complex waveform (such as the trace in Fig. 1D) can be reconstructed from a series of sine waves of different frequencies and amplitudes. The DFT identifies the characteristics of the sine waves required. 
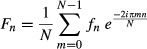


where *F*_*n*_ is the *n*th item in the output array.

*f*_*n*_ is the *n*th item in the input array.

*N* is the total number of samples.

*m* is the frequency where *m*/*N* is the samples per cycle.

*i* is the imaginary number corresponding to √−1.

*N* must equal an exponent of 2. This program is written to accept trains of 32 or 64 beats but could be adapted to analyze a greater number of beats. The theoretical maximum running Excel 2010 would be *N* = 2 (Lalani et al. 2013) or 8192 beats and 1,048,569 or almost 2 (Li et al. 2009) samples.

The DFT is calculated using the fast Fourier transform (FFT) algorithm. Although Microsoft Excel provides an inbuilt FFT algorithm, this runs slowly when called repeatedly. Instead, this program uses a different implementation of the originally described radix‐2 Cooley–Tukey FFT algorithm (Cooley and Tukey 1965) written for VBA. This is a divide‐and‐conquer algorithm that recursively splits a DFT of size N into smaller DFTs of sizes N/2. The particular adaptation used here is a slightly modified version of an algorithm written by Axel Vogt (www.axelvogt.de).

A DFT is calculated across all 32 beats for each timepoint. The output is in the form of a complex number – it has a real part comprising real numbers from −∞ to ∞ and an imaginary part comprising multiples of the imaginary number *i* (the square root of −1). The spectral magnitude of this complex number is calculated using equation 2

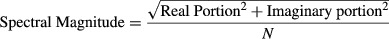


The spectral magnitude at each timepoint is stored in an output array. Spectral magnitude can be plotted against frequency to give a magnitude spectrum such as seen in Fig. 1E.

### Alternans quantification

In addition to defining an arbitrary minimum *V*_Alt_ (Fig. 1D), a decision as to whether alternans is present can be made using a measure of how much *V*_Alt_ exceeds random noise fluctuations. This is referred to as the *k*‐score and is calculated from equation 3.
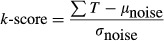


where ΣT is the spectral magnitude at 0.5 cycles/beat.

*μ*_noise_ is the mean spectral magnitude from 0.33 to 0.49 cycles/beat.

*σ*_noise_ is the standard deviation of the spectral magnitude from 0.33 to 0.49 cycles/beat.

A *k*‐score of three implies that *V*_Alt_ exceeds the noise floor by three standard deviations and is generally accepted as indicative of significant alternans (Verrier et al. 2011). It should be noted that *V*_Alt_ taken as the spectral magnitude at 0.5 cycles/beat is equivalent to half the difference between the mean of even beats and the mean of odd beats (Fig. 1D).

Equivalent measures of significance of 3:1 (*k*_3_‐score) and 4:1 oscillations (*k*_4_‐score) can be obtained in a similar manner. The *k*_3_ score can be calculated by employing ΣT as the spectral magnitude at 0.33 cycles/beat (or the mean of the closest two frequency bins) with the noise floor defined as frequencies from 0.25 to 0.5 cycles/beat excluding 0.33. Likewise, the *k*_4_ score uses ΣT as the spectral magnitude at 0.25 with the noise floor defined as frequencies from 0.26 to 0.49 cycles/beat. The noise floor in this case excludes 0.5 cycles/beat as 2:1 oscillations coexist with 4:1 oscillations as a harmonic.

### Action potential morphology and phase identification

**Figure 2.**
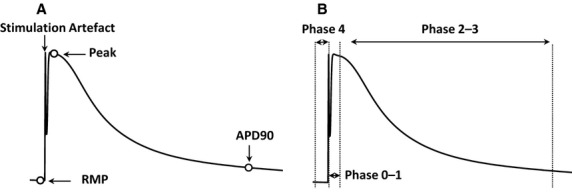
Action potential with (A) morphological features annotated (B) phases annotated.

A mean *E*_m_ at each timepoint is constructed leading to the formation of an average action potential. From this, various parameters are derived (Fig. 2A). The upstroke of the action potential is defined as the earliest timepoint that a threshold AP slope is reached. This is achieved by calculating the first derivative of the voltage trace and performing a linear search. A threshold d*V*/d*t* of 1*V*/*s* was found to be effective for transmembrane action potentials. The resting membrane potential (RMP) is defined as the mean *E*_m_ of the 10‐ms period prior to the start of the AP. The stimulation artifact is ignored by employing a blanking period after AP onset. The AP peak is defined as the most positive *E*_m_ following the blanking period using a maximum function. RMP and AP peak can also be manually identified by the user in cases of inappropriate automated detection in the presence of noise. The AP amplitude is derived from equation 4. 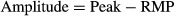


The *E*_m_ at 50% repolarization (*E*_m_APD_50_) and 90% repolarization (*E*_m_APD_90_) are derived from equation (5) 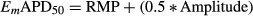


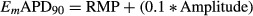


The times to 50% and 90% repolarization (APD_50_ and APD_90_) are defined as the time taken from the peak to *E*_m_APD_50_ and *E*_m_APD_90_, respectively.

The amplitude spectrum for each timepoint across the entire action potential can be averaged to give a measure of “whole trace” alternans. Alternans of specific regions of the AP can also be examined (Fig. 2) using parameters derived from morphological analysis of the mean AP.

The period of AP phases 0–1 (rapid upstroke to start of plateau) is defined here as from 5 ms prior to the AP peak to 5 ms after the peak. Phases 2–3 (start of plateau to end of repolarization) is defined here as from 5 ms after the AP peak to APD_90_ (90% repolarization). Phase 4 (diastole) is defined here as the period from 10 ms prior to AP upstroke to AP upstroke.

## Results

The user imports data from commercially available software such as Clampex (Molecular Devices) into the “Data input” window. Example data in this section were recorded from an ovine atrial myocyte using Clampex v10.2. Usual practice is to discard the first 10 beats after an abrupt rate change to minimize the chance of erroneously identifying short‐lived alternans which is physiologically normal. The user is prompted to enter demographic data, to be used during file saving. A drop‐down box is used to select whether 32 or 64 sweeps are to be analyzed. Analysis is initiated by clicking “Analyse Alternans”.

**Figure 3.**
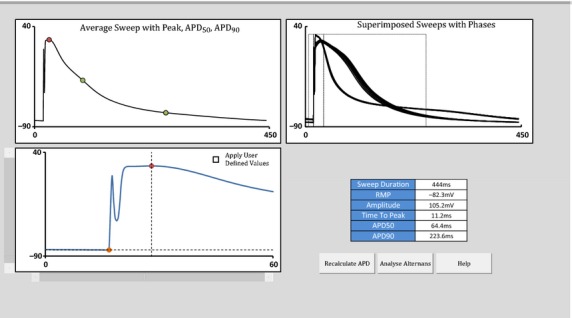
Screenshot of AP morphology window.

The next processing step involves analysis of AP morphology. This step can be bypassed if non‐AP data such as calcium transients is to be analyzed. The “AP morphology” window (Fig. 3) displays all beats superimposed (upper right pane). The mean AP is displayed with indicators highlighting the automatically determined AP peak, RMP, APD_50_, and APD_90_, having ignored the stimulation artifact (upper and lower left panes). The user can accept the calculated values or instead use scroll bars to manually adjust the position of Peak and RMP before recalculating. Automatically determined values can be reapplied by unchecking “Apply user defined values”. Once morphological characteristics have been successfully identified, clicking “Analyse Alternans” begins the Fourier Transform.

**Figure 4.**
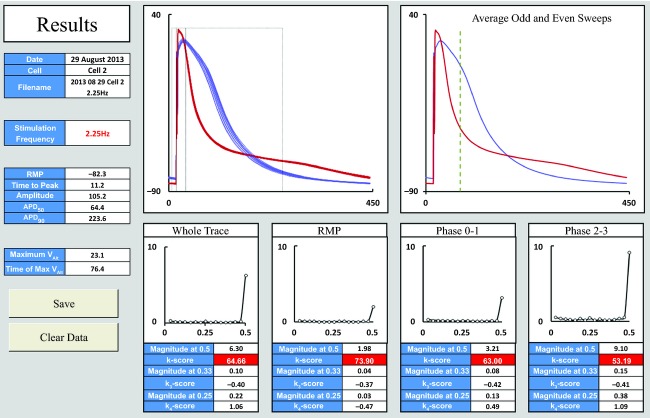
Screenshot of Results window.

The “Results” window (Fig. 4) contains several panes. The upper left pane displays superimposed beats with odd beats in red and even in blue. The calculated phases of the AP are marked for visual confirmation. The upper right pane shows the mean of odd beats and the mean of even beats. A dashed line represents the timepoint with maximal alternans. The lower panes show the magnitude spectra for each phase of the AP. Values for *V*_Alt_, *k*‐score, magnitude of 3:1 and 4:1 oscillations and *k*_3_ and *k*_4_‐scores are tabulated for each region. *K*‐scores showing significant alternans are highlighted in red.

Clicking “Save Data” causes a copy of the file to be saved to a path specified by the user in a <date> subdirectory. This facilitates logical storage and easy retrieval of data. Data are also copied to a “Collated results” window. While saving and clearing data will wipe data from the “Data input”, “AP morphology” and “Results” windows, the “Collated data” window retains each run of the program in a new row. This enables easy export of a batch of results at the end of an analysis session.

### Examples

**Figure 5.**
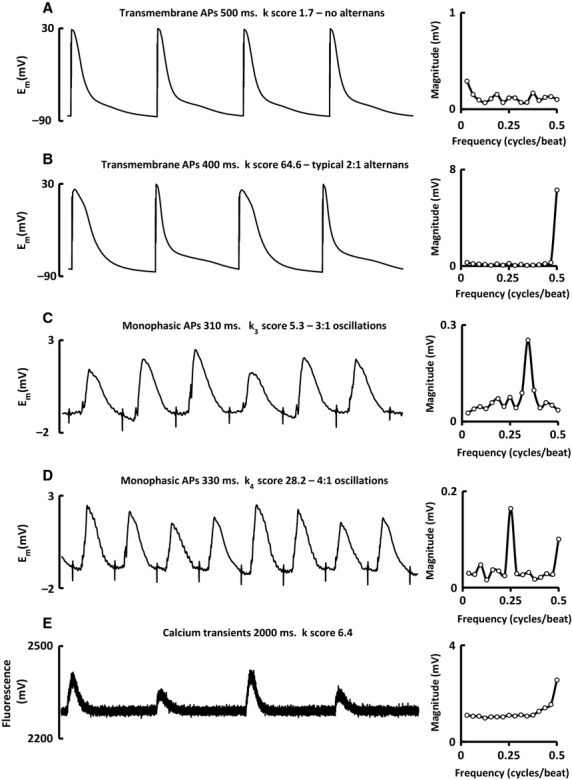
Examples of representative traces with their corresponding magnitude spectra. (A) Transmembrane APs showing no alternans. (B) Transmembrane APs showing alternans. (C) Monophasic APs showing 3:1 oscillations. (D) Monophasic APs showing 4:1 oscillations. (E) Calcium transients showing alternans. APs – Action potentials.

The software is optimized to analyze action potentials which can be recorded using a variety of methods, although other biological traces can also be analyzed. Figure 5A and B show recordings from ovine atrial transmembrane action potentials recorded using the perforated patch‐clamp technique along with their whole trace magnitude spectra. The first shows no alternans as indicated by the essentially flat magnitude spectrum, while the second shows typical alternans behavior and a corresponding peak at 0.5 cycles/beat.

Figure 5C and D show endocardial atrial monophasic action potentials recorded from an anesthetized sheep. Figure 5C shows 3:1 oscillations made clear by the peak at 0.33 cycles/beat, while Fig. 5D shows 4:1 oscillations with peaks at 0.25 and 0.5 cycles/beat.

Figure 5E is a recording of cytosolic calcium concentrations using the fluorescent dye Fluo‐5F. It should be noted that this recording was performed at a low stimulation frequency with an inter‐pulse interval of 2000 ms. This has resulted in a long baseline period which is identical between large and small calcium transients. Averaging alternans across the whole trace means that the apparent magnitude of oscillations will be diminished if the alternating proportion of the trace is small.

### Accuracy in presence of noise

**Figure 6.**
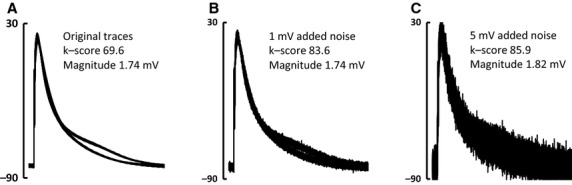
Alternans detection and quantification remains robust despite the addition of noise. (A) Original trace – 32 superimposed atrial action potentials demonstrating repolarization alternans. (B) Traces seen in A with 1 mV RMS of Gaussian noise added. (C) Traces seen in A with 5 mV of Gaussian noise added.

Although the action potentials displayed in Fig. 5 have a good signal‐to‐noise ratio, the software can still function well when analyzing signals contaminated with electrical noise. Figure 6A shows 32 superimposed transmembrane action potentials with low amplitude alternans present. Figure 6B shows the same traces with 1 mV RMS Gaussian noise added to each sample. Analysis produces similar values of k‐score and *V*_Alt_. Figure 6C shows the same traces with 5 mV of added Gaussian noise. Similar results were obtained when testing on data from monophasic action potential recordings showing very subtle alternans. Reliable detection of alternans was achieved with noise levels up to 5‐fold greater than *V*_Alt_ when 32 beats were analyzed. Resilience to noise increased when 64 sweeps were analyzed so that accurate detection of alternans was possible when noise exceeded *V*_Alt_ by a factor of 10. Quantification of alternans was within 10% of initial values in the presence of noise that was one‐fold and six‐fold greater than *V*_Alt_ when 32 and 64 beats were used, respectively. At the highest noise levels that still permitted alternans identification, calculated *V*_Alt_ was within 50% of the true value.

These results are broadly comparable with previous data that assessed the reliability of the spectral method when used to analyze EGM T‐wave alternans in the presence of noise (Orini et al. 2014)_._

## Discussion

Alternans has been studied from the whole heart to the subcellular level. A corresponding variety of techniques have been applied to quantify alternans behavior. These range from simple methods of calculating the ratios of amplitude or APD_90_ for odd to even beats (Li et al. 2009; Wan et al. 2012) to highly sophisticated mathematical approaches (Monasterio et al. 2009). The most commonly applied techniques in the field of T‐wave alternans analysis are the spectral method and the modified moving average approach as these are built into commercially available hardware. Other reported methods include the Laplacian likelihood ratio, complex demodulation, and Monte Carlo simulation (Nearing and Verrier 2002; Iravanian et al. 2012; Orini et al. 2014).

Several studies have compared techniques for analyzing alternans but each has been with reference to a specific signal type: T‐wave alternans on the surface ECG (Burattini et al. 2009), repolarization alternans of epicardial electrograms (Orini et al. 2014), or alternans of optically recorded APs (Iravanian et al. 2012). The different characteristics of each signal type studied make overall generalizations regarding the overall superiority of a particular technique problematic. The spectral method typically performs well in cases of wideband noise and low‐frequency amplitude oscillations such as those due to respiration (Burattini et al. 2009; Orini et al. 2014). The spectral method may be suboptimal when used to assess alternans that is nonstationary, that is, that changes in magnitude or disappears during parts of a recording. Accuracy particularly suffers during instances of phase change due to dropped beats or ectopic beats, in which case Monte Carlo‐based algorithms may offer an advantage (Iravanian et al. 2012). This may be more of a problem for long recordings of 128 beats or more such as during the assessment of T‐wave alternans (Armoundas et al. 2012). Traditional implementations of all these techniques ignore non‐2:1 oscillations which may be of even greater significance in the initiation of arrhythmias than classical alternans (Nearing and Verrier 2002).

The spectral method of alternans analysis applied here is therefore most suitable for relatively short recordings (32 or 64 beats) that are least likely to experience dropped beats, phase changes, or other nonstationarities. It is robust in the presence of noise or baseline drift due to respiration. Unlike other methods, higher order periodicities are not ignored. The facility to examine depolarization and repolarization alternans independently presents an additional advantage.

## Specifications and mode of availability

This program will run on Microsoft Excel 2010 or 2013. Using a Dell Optiplex 745 with an Intel Core2 6400 CPU running at 2.13GHz with 3.25GB of RAM, processing time for 32x 5000 samples took 2.5 s for AP morphology analysis and 4 s for alternans quantification. File size is approximately 250 kB. A copy of the program can be obtained by emailing the author.

## Conclusions

Action potentials alternans is an important electrical observation that is linked to cardiac arrhythmias and sudden cardiac death. This study describes the first complete and self‐contained tool to perform spectral analysis of this widely studied phenomenon and be made publicly available. It is written in VBA and runs within the Microsoft Excel environment. Robust discrimination between alternans and nonalternans is performed, the magnitude of alternans is quantified, regionality is determined and higher order oscillations are identified. This is coupled with an analysis of action potential morphology including amplitude, resting membrane potential and repolarization times. Parameters are analyzed rapidly, presented alongside easily appreciable graphical representations. Successive program runs are saved in an easily exportable collated format.

## Acknowledgments

I would like to thank Professor David Eisner, Dr Katharine Dibb, and Professor Andrew Trafford for their input and support. I would also like to thank Dr David Greensmith and Dr Graeme Kirkwood for their advice and encouragement.

## Conflict of Interest

The author has no conflicts of interest to declare.
